# The key regulator *LcERF056* enhances salt tolerance by modulating reactive oxygen species-related genes in *Lotus corniculatus*

**DOI:** 10.1186/s12870-021-03336-4

**Published:** 2021-12-29

**Authors:** Dan Wang, Zhanmin Sun, Xinxu Hu, Junbo Xiong, Lizhen Hu, Yuandong Xu, Yixiong Tang, Yanmin Wu

**Affiliations:** 1 Biotechnology Research Institute, Chinese Academy of Agricultural Sciences, Beijing, China; 2 Key Laboratory of Southwest China Wildlife Resources Conservation (Ministry of Education), China West Normal University, Nanchong, China; 3 College of Animal Science and Technology, Hunan Agricultural University, Changsha, China; 4 Hubei Key Laboratory of Animal Embryo and Molecular Breeding, Institute of Animal and Veterinary Science, Hubei Academy of Agricultural Science, Wuhan, China; 5 Institute of Animal and Veterinary Science, Jiangxi Academy of Agricultural Sciences, Nanchang, China; 6 ChongQing Academy of Animal Sciences, Chongqing, China; 7 Huanshan group, Qingdao, China

**Keywords:** ERF transcription factor, Salt stress, Regulation mechanism, *Lotus corniculatus*

## Abstract

**Background:**

The APETALA2/ethylene response factor (AP2/ERF) family are important regulatory factors involved in plants’ response to environmental stimuli. However, their roles in salt tolerance in *Lotus corniculatus* remain unclear.

**Results:**

Here, the key salt-responsive transcription factor *LcERF056* was cloned and characterised. *LcERF056* belonging to the B3–1 (IX) subfamily of ERFs was considerably upregulated by salt treatment. LcERF056-fused GFP was exclusively localised to nuclei. Furthermore, *LcERF056*- overexpression (OE) transgenic *Arabidopsis* and *L. corniculatus* lines exhibited significantly high tolerance to salt treatment compared with wild-type (WT) or RNA interference expression (RNAi) transgenic lines at the phenotypic and physiological levels. Transcriptome analysis of OE, RNAi, and WT lines showed that *LcERF056* regulated the downstream genes involved in several metabolic pathways. Chromatin immunoprecipitation-quantitative polymerase chain reaction (ChIP-qPCR) and yeast one-hybrid (Y1H) assay demonstrated that LcERF056 could bind to cis-element GCC box or DRE of reactive oxygen species (ROS)-related genes such as lipid-transfer protein, peroxidase and ribosomal protein.

**Conclusion:**

Our results suggested that the key regulator *LcERF056* plays important roles in salt tolerance in *L. corniculatus* by modulating ROS-related genes. Therefore, it may be a useful target for engineering salt-tolerant *L. corniculatus* or other crops.

**Supplementary Information:**

The online version contains supplementary material available at 10.1186/s12870-021-03336-4.

## Background

Salinity is one of the most widespread abiotic stresses that limits plant growth and crop productivity. Approximately 7% of the land area and 19.5% of arable lands worldwide are under salt stress [1]. Salt exerts detrimental effects on plants by causing osmotic stress, ion imbalance, and oxidative toxicity. To survive salt stress, plants have evolved to have high plasticity and elaborate mechanisms, comprising osmotic stress resistance, ion exclusion, and tissue tolerance. The stress-related molecular regulatory network is complex and mostly unexplored [2]. Hence, exploring the key and novel genes that regulate salt stress-related genes has become increasingly important in modern agriculture.

As key regulators, transcription factors (TFs) have crucial functions in plant growth and development and environmental stress responses. The APETALA2/ethylene responsive factor (AP2/ERF) is one of the most important families of plant-specific TFs. It comprises a well-conserved AP2/ERF domain consisting of 50–70 amino acid residues, and it binds to some specific *cis*-acting elements such as dehydration-responsive element (DRE) and GCC box for the regulation of expression of many downstream genes [3].

In recent years, many AP2/ERF genes involved in inducing abiotic or biotic stress response have been reported. For instance, *IbRAP2–12*, from the salt-tolerant sweet potato, was induced by NaCl, PEG, ABA, and MeJA. *IbRAP2–12*-overexpressing *Arabidopsis* lines were found to be more tolerant to salt and drought stresses than wild-type (WT) plants [4]. Overexpression of *GsERF7* and *GsSnRK1* synergistically increased the tolerance of transgenic *Arabidopsis* plants to salt–alkali stress [5]. Overexpression of *ERF1-V* in wheat could improve resistance to powdery mildew, salt, and drought stress [6]. In addition, AP2/ERF TFs have been found to have close association with plant growth and development and hormone responses. *OsERF48* was reported to enhance root growth and drought tolerance by regulating a calmodulin-like protein gene *OsCML16* [7]. *Arabidopsis* overexpressing *AtERF019* was reported to have a 7-day delay in flowering, a 2-week delay in senescence, and increased tolerance to water deprivation when compared with WT plants [8]. Rice ethylene-response AP2/ERF factor *OsEATB* can restrict internode elongation by downregulating a gibberellin biosynthetic gene [9]. These results indicated that the AP2/ERF genes exhibit various functions and play crucial roles in the growth, development, and biotic or abiotic stress responses in plants.

*L. corniculatus* (Birds foot trefoil), a member of perennial legume forage, is considered to be one of the most agriculturally important forage plants due to their major agricultural advantages, including the anti-bloating properties because of tannin content and ability to grow in low fertile, acidic, and highly saline soils, and it is widely used to prevent roadside erosion. Previously, we isolated and identified 127 AP2/ERF genes from *L. corniculatus*. Expression profile analysis of these AP2/ERF genes by quantitative real-time PCR (qPCR) revealed that 19 LcERF genes were significantly upregulated by salt stress. Among these genes, *LcERF054*, *LcERF080*, and *LcAP2/ERF107* were found to significantly enhance the tolerance to salt stress in transgenic *Arabidopsis* plants [10–12]; however, the molecular mechanism remains unclear.

To understand the molecular mechanisms underlying salt tolerance in *L. corniculatus* in detail, we conducted the characterisation and function analysis of *LcERF056* and further assessed the mechanisms of action and regulatory pathways that affect salt stress response in *L. corniculatus*.

## Results

### Molecular characteristics of *LcERF056*

The full-length cDNA of *LcERF056* (GenBank accession KC777345) was isolated from *L. corniculatus*. The gene contained a 558-bp open-reading frame and encoded a protein with 185 amino acid residues. Additionally, genomic DNA containing the 5′ and 3′ untranslated regions of *LcERF056* was sequenced. No introns were found in the genomic *LcERF056* upon comparison with the cDNA and genomic DNA sequences (data not shown). Analysis of the amino acid sequence of LcERF056 showed that the protein contained a single AP2/ERF DNA-binding domain (amino acids 121–179) (Fig. S1A). Phylogenetic tree (Fig. S1B) analysis suggested that *LcERF056* was closely related to *AtERF013* (with a 35.55% sequence identity) and belonged to the B3–1 (IX) group of ERF transcription factor family (Fig. S1C).

### Expression pattern of *LcERF056*

**Fig. 1:**
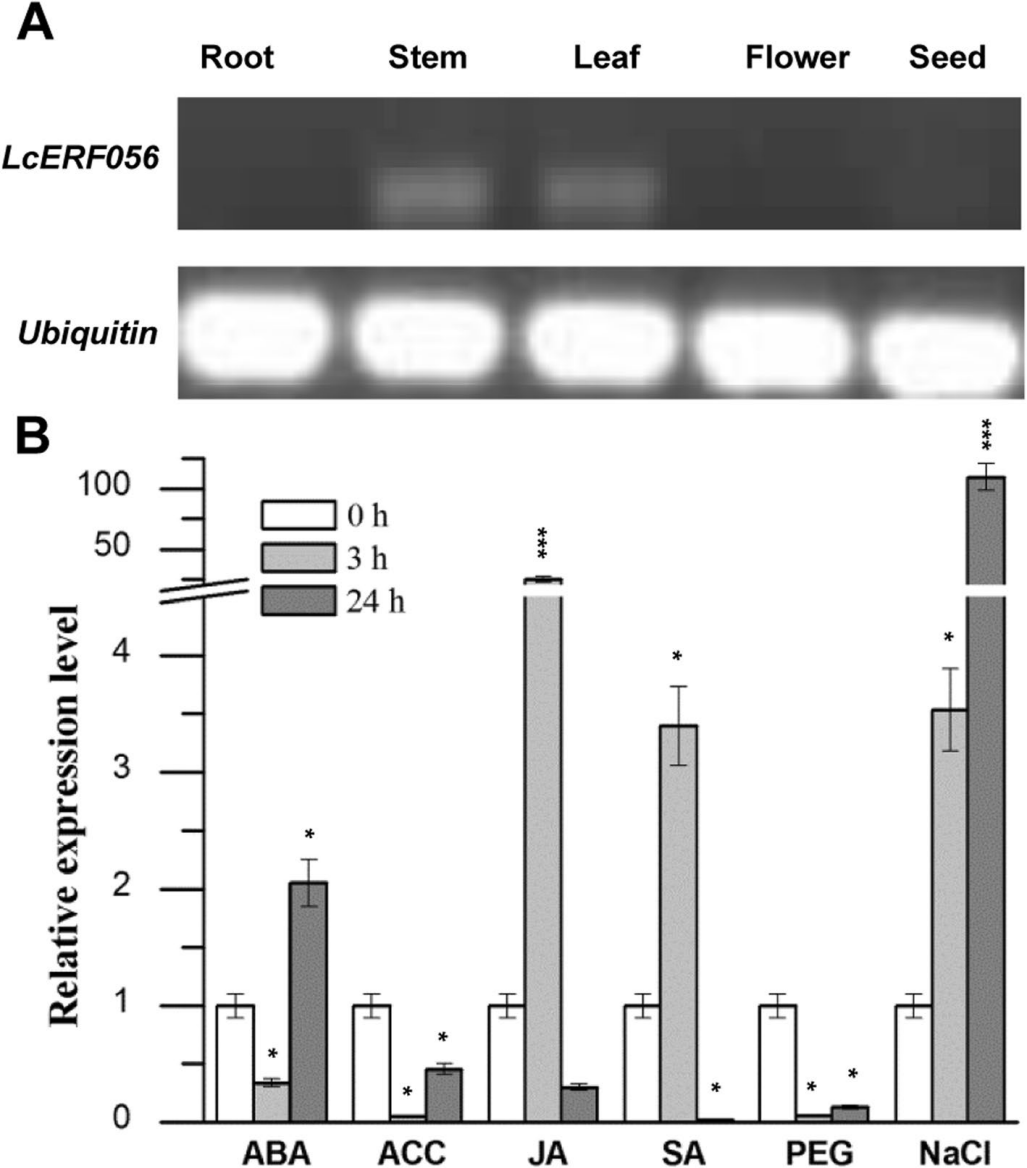
The expression patterns of *LcERF056* mRNA. **(A)** Expression of *LcERF056* mRNA in various tissues evaluated using RT-PCR. **(B)** Expression level of *LcERF056* in response to abiotic stresses. Four-week-old seedlings were exposed to 100 μM of ABA, ACC, JA, SA, 20% PEG, or 150 mM NaCl. The relative expression was calculated using *polyubiquitin* as an internal reference. The WT unstressed expression level was assigned a value of 1. Data represent the averages of three independent experiments ± SE

To investigate the spatial expression patterns of *LcERF056*, RT-PCR was performed with mRNAs extracted from different organs, including roots, stems, leaves, flowers, and seeds of *L. corniculatus*. *LcERF056* was found to express in the stems and leaves, whereas its expression was not detected in the roots, flowers, or seeds (Fig. 1A). To study the effects of different stress signals on gene expression, *L. corniculatus* seedlings were treated with ABA, ACC, JA, SA, PEG, or NaCl. RNA extracted from the seedlings at 0, 3, and 24 h after the treatments were subjected to qPCR to evaluate the mRNA levels of *LcERF056* upon exposure to various stress signals. The results (Fig. 1B) showed that mRNA of *LcERF056* could be detected in all cases after 3 and 24 h. In seedlings treated with NaCl (24 h), *LcERF056* was significantly upregulated (110-fold higher than the WT). Additionally, JA treatment could increase the mRNA levels by 24-fold at 3 h, whereas ABA and SA treatments exhibited only a slight effect on the mRNA levels of *LcERF056*. After ACC and PEG treatments, *LcERF056* mRNA levels were found to decrease significantly at 3 and 24 h.

### LcERF056 localises to the nucleus and acts as a transcriptional activator

**Fig. 2:**
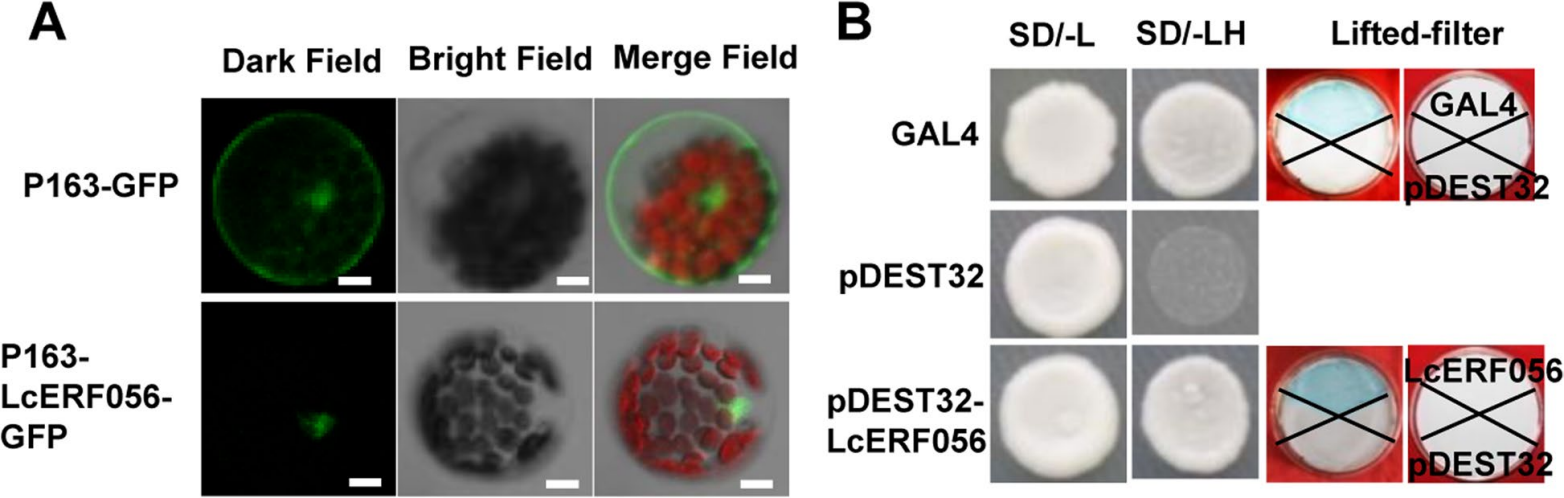
LcERF056 is a nuclear protein acting as a transcriptional activator. **(A)** Subcellular localisation of LcERF056. Plasmids containing 35S:GFP and 35S:LcERF056-GFP were introduced into *Arabidopsis* protoplast by polyethylene glycol transfection. Nuclear localisation was investigated through confocal microscopy. Bars = 5 μm. **(B)** Analysis of transcriptional activation of LcERF056 by the yeast one-hybrid assay

To examine the subcellular localisation of LcERF056, *LcERF056* was fused to the green fluorescent protein (GFP) gene driven by CaMV35S promoter. The fused gene 35S:LcERF056-GFP was transiently expressed in *Arabidopsis* protoplast. As a control, similar experiment without *LcERF056* fusion (GFP gene only) was conducted at the same time. LcERF056-GFP was found to be localised in the nucleus, whereas the control GFP was distributed throughout the cells (Fig. 2A). Further, to understand the function of LcERF056, yeast one-hybrid (Y1H) assay was performed using the construct carrying *LcERF056.* Results of the Y1H assay demonstrated that LcERF056 could act as an activator for the transcription of genes (Fig. 2B).

### Overexpression of *LcERF056* in *Arabidopsis* enhances salt tolerance

**Fig. 3:**
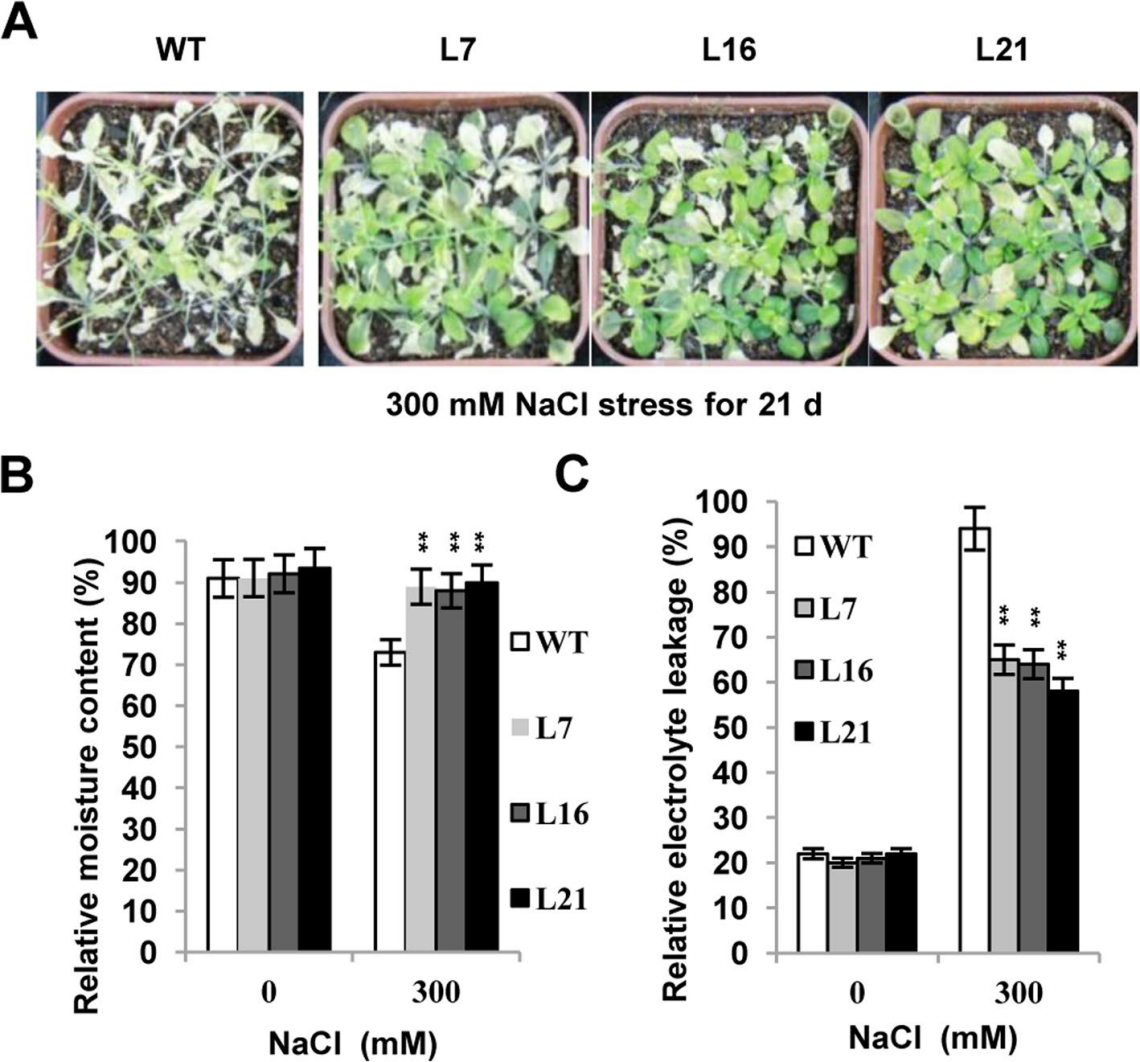
Overexpression of *LcERF056* confers enhanced salt tolerance in transgenic *Arabidopsis*. **(A)** Growth of WT and transgenic plants under 300 mM NaCl stress after 21 days. Ten-day-old seedlings grown on MS medium plates were planted in pots filled with a 1:1 mixture of vermiculite and humus and well-watered for 20 days. The seedlings were watered with a solution containing 300 mM NaCl for 21 days. The experiment was repeated three times. **(B)** Relative moisture contents. **(C)** Relative electrolyte leakage. The data represent the means of three replicates. The error bars indicate the SD. **P < 0.01 compared with the WT plants, using Student’s t test

No obvious morphological difference was observed between the WT plants and all the 10 lines of *35S:LcERF056* transgenic *Arabidopsis* plants. However, the 4-week-old *35S:LcERF056* plants exhibited higher tolerance to salt stress than the WT after they were treated with NaCl for 21 days (Fig. 3A). The relative moisture content was lower in WT than in the transgenic plants (Fig. 3B). The relative membrane ion leakage, an index for the level of cellular damage, was significantly lower in *35S:LcERF056* plants than in WT plants (Fig. 3C). The results suggested that *LcERF056* was involved in defence of salt stress, and ectopic expression of *LcERF056* could increase the salt tolerance in *Arabidopsis*.

### *LcERF056* enhances defence against salt stress in *L. corniculatus*

To explore the endogenous function of *LcERF056* in *L. corniculatus*, *LcERF056* knockdown experiments were conducted. For comparison, *LcERF056* was overexpressed in *L. corniculatus* at the same time. RNA interference plants (*LcERF056*-RNAi) and overexpressing plants (*LcERF056*-OE) of *L. corniculatus* were obtained through *Agrobacterium*-mediated transformation. The presence of the transgenes in the putative transgenic *L. corniculatus* was examined through PCR genotyping. As shown in Fig. S2, the transcript of *LcERF056* greatly decreased in the *LcERF056*-RNAi lines and increased significantly in the *LcERF056*-OE lines compared with that in WT plants. The phenotypes were characterised using in vitro tissue culture plants (Fig. S3) because no seeds could be obtained when *LcERF056*-OE plants were grown in the laboratory or greenhouse. The *LcERF056*-OE lines exhibited a significantly lower plant height but more branches than the WT at 90 days, whereas a significantly higher plant height and fewer branch numbers were observed in *LcERF056*-RNAi lines (Fig. S3).

**Fig. 4:**
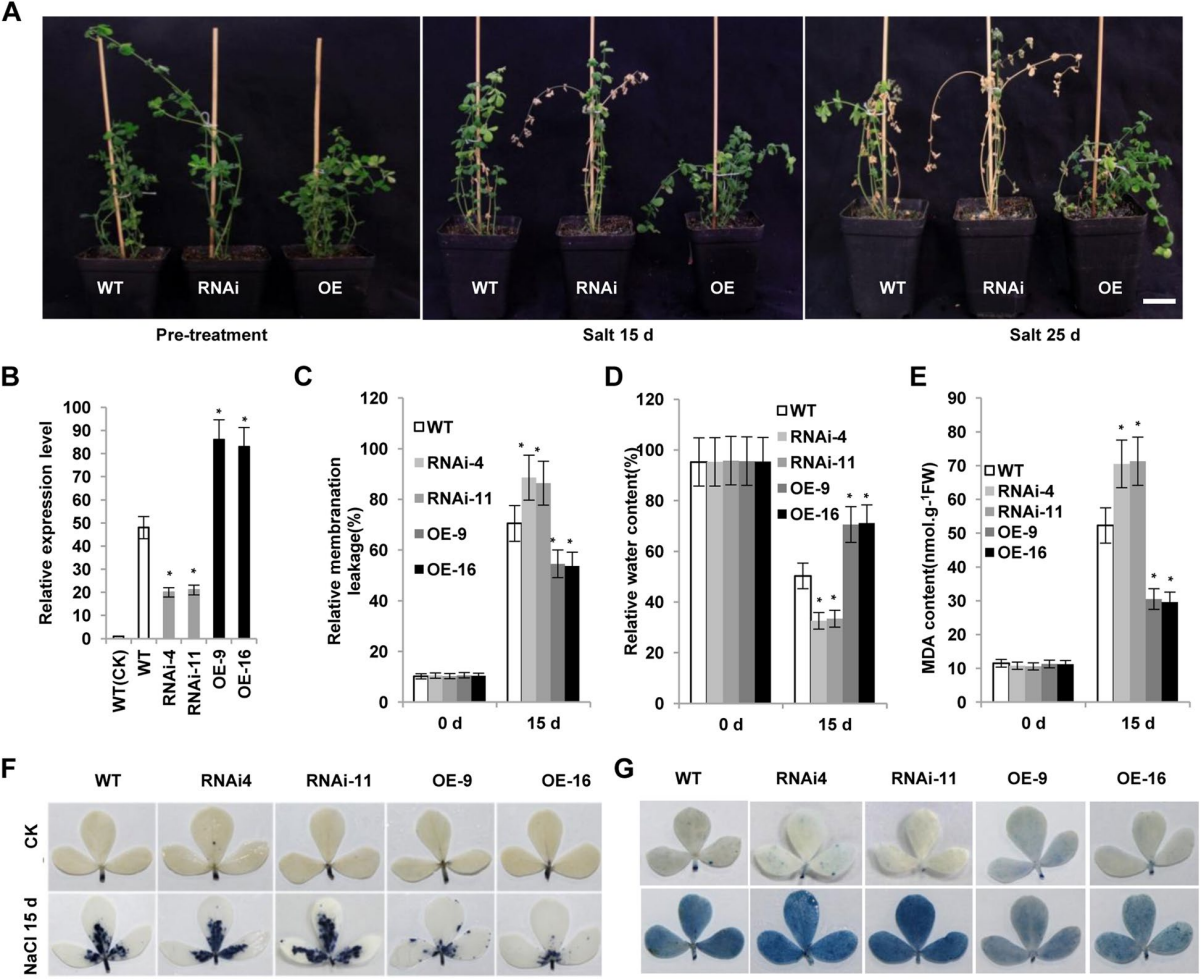
Changes in phenotypic and physiological traits in transgenic *L. corniculatus* under salt stress. **(A)** Growth of WT and transgenic plants under 250 mM NaCl stress after 15 and 25 days. Bars = 5 cm. **(B)** Relative expression level of *LcERF056* in the WT and transgenic lines under 250 mM salt stress. **(C)** Relative membrane ion leakage. **(D)** Relative water content. **(E)** MDA content. **(F)** Trypan blue staining. **(G)** Nitroblue tetrazolium staining. *P < 0.05 between WT and transgenic line plants, using the Student’s t test

To examine whether *LcERF056* is involved in defence against salt stress in *L. corniculatus*, we treated WT, *LcERF056*-RNAi, and *LcERF056*-OE plants, which had been transplanted into pots for 10 days in advance, with 250 mM NaCl solution. Salt stress considerably inhibited the plant growth (Fig. 4A). After 15 days, approximately half of the leaves of *LcERF056*-RNAi plants turned yellow, whereas the leaves of WT and *LcERF056*-OE lines remained green. The *LcERF056-*RNAi plants died after 25 days; however, only some of the leaves of the WT plants turned yellow and those of the *LcERF056*- OE plants remained green (Fig. 4A). Further analysis showed that the membrane ion leakage increased significantly in the *LcERF056*-RNAi lines and decreased in the *LcERF056*-OE lines in comparison with the WT plants (Fig. 4C). Other salt-stress-tolerance indices, such as lipid peroxidation levels represented by malondialdehyde (MDA) content, relative water content, accumulation of superoxide ion superoxide (O_2_^−^), and cell death inferred by trypan blue staining (Fig. 4D–G), were assessed. Under salt stress, *LcERF056*-RNAi plants showed significantly lower water content, higher MDA content, higher accumulation of O_2_^−^, and enhanced cell death. Conversely, *LcERF056*-OE plants exhibited significantly higher water content, lower MDA content, lower accumulation of O_2_^−^, and lower cell death (Fig. 4D–G). The results suggested that *LcERF056* played a crucial role in salt stress defence in *L. corniculatus.*

### *LcERF056* overexpression leads to extensive transcription of stress-responsive genes

To investigate the underlying molecular mechanism of salt stress defence mediated by *LcERF056*, RNA-Seq of the WT, *LcERF056*-RNAi, and *LcERF056*-OE plants was performed. Differential expression analysis identified 47 upregulated and 80 downregulated genes (P < 0.05) in *LcERF056*– RNAi plants compared with the WT plants. In *LcERF056*-OE plants, 44 upregulated and 76 downregulated genes were detected (Fig. S4A). Results of the gene ontology (GO) analysis suggested that these genes could regulate multiple biological processes, including regulation of transcription, plant response to stresses, and growth (Fig. S4B, C).

**Fig. 5:**
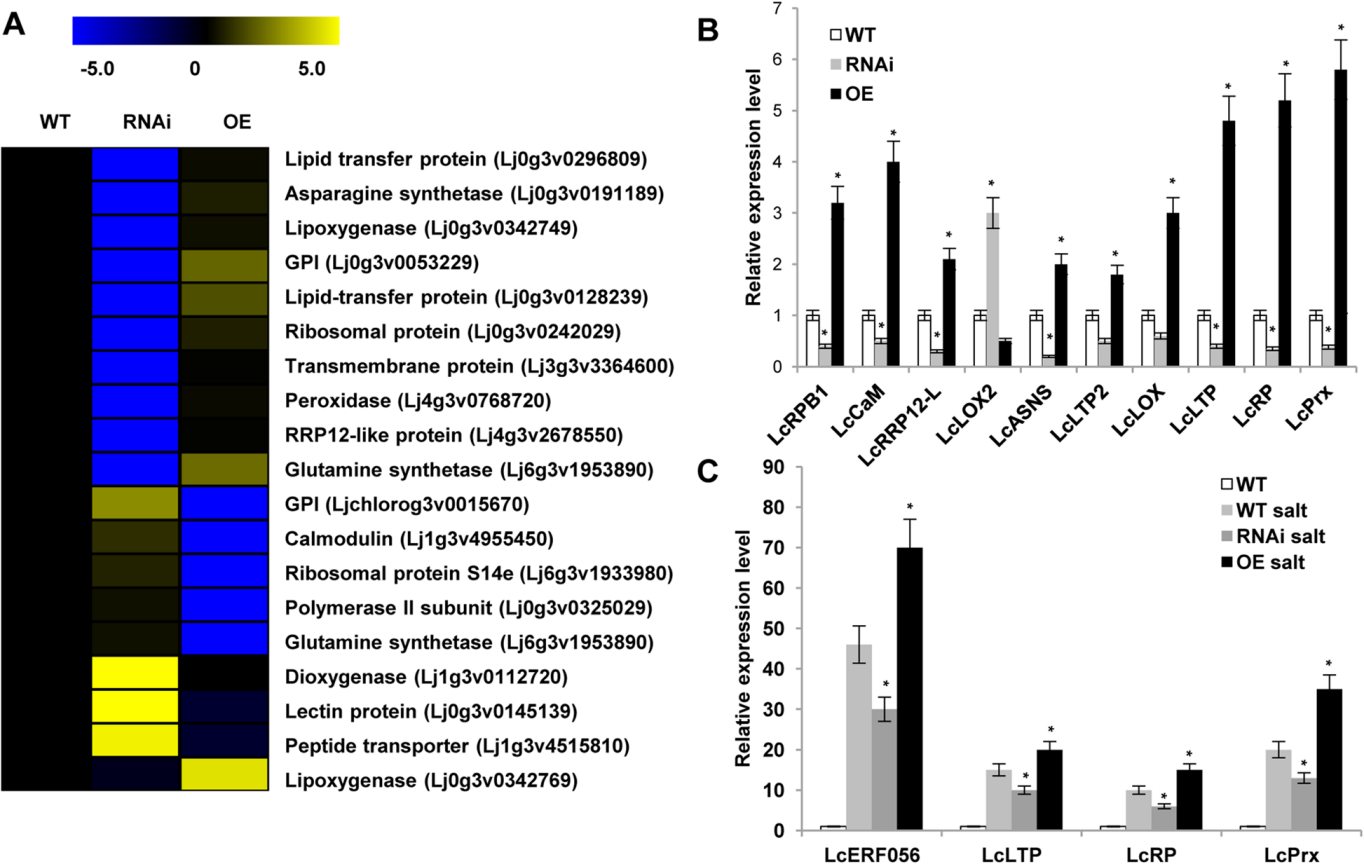
Differentially expressed genes in WT, *LcERF056*-OE, and *LcERF056*-RNAi transcriptome. **(A)** Heatmap of 19 genes related to growth and stress. **(B)** Expression patterns of 10 genes related to growth and stress in WT, *LcERF056*-OE, and *LcERF056*–RNAi lines. **(C)** The relative expression level of the 3 target genes in WT, *LcERF056*-OE, and *LcERF056*-RNAi lines under salt treatment. The WT expression level was assigned a value of 1. Data represent the averages of three independent experiments ± SE

To verify the candidate target genes, expression levels of the overlapped 18 genes (Table S3, S4) were analysed. Ten out of the 18 genes were further selected for qPCR analysis using *L. corniculatus ubiquitin* as the reference gene. The measured expression levels of most genes were highly consistent with the levels determined by RNA-Seq (Fig. 5A, B).

### Identification and validation of LcERF056-regulating target genes by direct binding to motifs by using yeast one-hybrid (YIH) assay and ChIP-qPCR

Promoters of the aforementioned 10 DEGs were isolated and analysed by PlantCARE. Promoters of three genes, *LcLTP*, (lipid-transfer protein, LTP, Lj0g3v0128239), *LcRP* (ribosomal protein, RP, Lj0g3v0242029), and *LcPrx* (peroxidase, Lj4g3v0768720), were found to have the DRE element or GCC box (Table S6). Expressions of *LcLTP*, *LcPrx*, and *LcRP* in *LcERF056*-OE and *LcERF056*-RNAi plants under salt stress were analysed using qPCR. For comparison, the same experiment was conducted using the WT line as control. The results indicated that *LcLTP*, *LcRP*, and *LcPrx* were significantly downregulated in *LcERF056*-RNAi plants and highly upregulated in *LcERF056*-OE plants, consistent with the expression patterns of the *LcERF056* gene (Fig. 5C). These three genes are known to be closely related to salt stress. Lipid-transfer proteins (LTPs), a class of small, ubiquitous proteins, play critical roles in various environmental stresses [13]. *RP* genes have been shown to be differentially regulated by abiotic and biotic environmental factors [14, 15]. Peroxidase plays prominent roles in antioxidant responses and stress tolerance in plants [16].

**Fig. 6:**
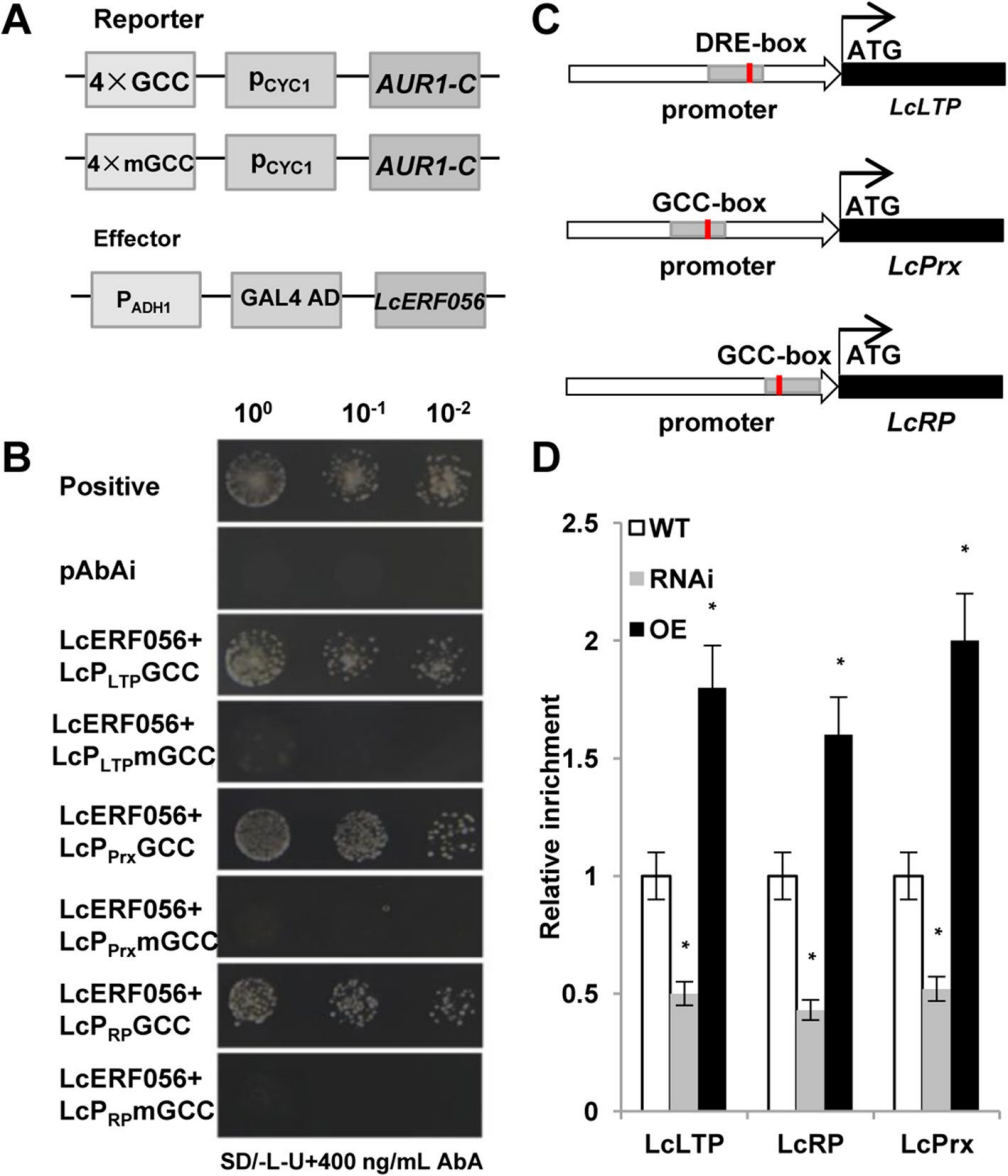
LcERF056 binds directly to GCC-box and DRE motifs in promoters to activate the transcription of downstream target genes. **(A)** Reporter and effector vectors used for yeast one-hybrid (YIH) experiments. **(B)** Verification of the interactions between LcERF056 and GCC-box and DRE cis elements through YIH experiments. Positive transformants were identified by spotting serial dilutions of yeast (1:1, 1:10, and 1:100) onto SD/−L-U plates supplemented with 400 ng/mL AbA. **(C)** Schematic structure of *LcLTP*, *LcRP*, and *LcPrx* genes. ATG, translation start point; Red line, GCC-box or DRE-box; Grey boxes, ChIP-qPCR amplified fragments that correspond to the regions indicated in the gene structure. **(D)** ChIP-qPCR analysis of the enrichment levels of *LcLTP*, *LcRP*, and *LcPrx* in *LcERF056*-OE and *LcERF056*-RNAi transgenic plants by using the specific synthetic antibody (rabbit). WT assay was included as control. *P < 0.05 between WT and transgenic plants, using Student’s t test

To study whether LcERF056 could directly bind to the promoters of these genes, Y1H assay of *LcERF056* was performed. The results showed that yeast cells harbouring *LcERF056* and GCC box grew well in the presence of aureobasidin A (AbA), whereas cells with mutated GCC box could not survive (Fig. 6A, B). To further assess the interactions between LcERF056 and the GCC box in vivo, LcERF056 antibody was produced in advance in the present study. Proteins were extracted from *L. corniculatus* and subjected to western blotting. Only a single band around 22.2 kDa was observed for each sample (Fig. S5), demonstrating the specificity of the LcERF056 antibody. The chromatin immunoprecipitation (ChIP) qPCR revealed that the *LcLTP*, *LcRP*, and *LcPrx* promoter enrichment fold were significantly higher in the *LcERF056*-OE plants than in the WT or *LcERF056*-RNAi plants (Fig. 6C, D). These observations suggested that the binding of LcERF056 on the promoters of *LcLTP*, *LcPrx*, and *LcRP* was positively correlated with its transcript levels.

## Discussion

### *LcERF056* is the key salt-responsive TF in *L. corniculatus*

AP2/ERF is one of the most important families of plant-specific TFs responsible for plant growth, development, and abiotic or biotic stress response [17, 18]. This superfamily consists of several members, and to date, over 14 billion members have been identified in *Arabidopsis*, rice, soybean, and *L. corniculatus* [10, 19, 20]. However, the members playing a critical role in response to salt stress have not been identified yet. Thus, identifying the essential salt-responsive genes and investigating the underlying mechanisms are essential.

Overall, 127 *AP2*/*ERF* genes have been identified from *L. japonicus* genome [10]. To investigate if these *L. corniculatus AP2*/*ERF* genes are involved in defence against salt stress, we analysed the expression of 106 *AP2*/*ERF* genes in response to salt treatment. Nineteen of these *AP2*/*ERF* genes exhibited differential expression (fold change > 2, *P* < 0.05). Among them, *LcERF078* and *LcERF056* exhibited 300- and 110-fold higher expression, respectively, which indicated that these two genes are potentially involved in defence against salt stress*.* The 19 *AP2*/*ERF* genes were cloned into a binary vector driven by CaMV35S promoter and transferred into *Arabidopsis*, and independent transgenic lines for the 19 genes were obtained. Because the seeds yielded by the T1 generation of transgenic line expressing *LcERF078* could not yield normal seeds, no further experiments were performed on *LcERF078* in this study. Transgenic *Arabidopsis* plants overexpressing the other 18 genes could yield seeds normally, and no obvious morphological differences were observed in the aerial tissues as compared with the WT plants.

After treatment with NaCl for 21 days, the 4-week-old *LcERF056*-OE *Arabidopsis* plants exhibited marked tolerance to salt stress as compared with the WT (Fig. 3). Results of further experiments with *LcERF056*-OE and *LcERF056*-RNAi *L. corniculatus* plants revealed that the expression of *LcERF056* could enhance salt tolerance in *L. corniculatus*, thus suggesting that *LcERF056* is one of the key salt-responsive TFs in *L. corniculatus*.

### *LcERF056* probably affects plant development

In addition to the observation that *LcERF056* was involved in response to salt stress, expression of *LcEFR056* was negatively correlated with the plant height and positively correlated with branching number in *L. corniculatus* (Fig. S3). *LcERF056*-OE transgenic *L. corniculatus* lines exhibited dwarf phenotypes, indicating that the internodal elongation process was suppressed by *LcERF056* overexpression. Similar phenomenon appeared in rice AP2/ERF factor *OsEATB.* Qi has reported that *OsEATB* could restrict internode elongation by downregulating a gibberellin biosynthetic gene [9], whereas, Agarwal et al. suggested that the *AtERF11* TF promoted internode elongation by activation of gibberellin biosynthesis and signalling [21]. Bajaj identified that *ERFs* were one of the candidate genes for dissecting complex branch number trait in chickpea [22]. Plant shoot branching and height are important traits that affect forage grass production. However, there is no evidence that *LcERF056* directly regulates the downstream factors of GA biosynthetic gene. Further studies are required to assess the roles of *LcERF056* in internodal elongation and branch number.

### *LcERF056* enhanced salt tolerance by binding to the *cis*-element of ROS-related target genes

ERF subfamily genes play various roles in response to various environmental stress factors [8, 23, 24]. Previous studies have established that the AP2/ERF family regulates the transcriptional expression of downstream genes by binding to GCC box or DRE element [25, 26]. Our RNA-Seq data revealed that *LcERF056* regulated the expression of many genes related to biotic stress. Additionally, we analysed the *cis*-element in the promoters of the candidate genes and verified that LcERF056 could bind directly to promoters of *LcLTP*, *LcRP*, and *LcPrx*. Using tblastn programme at website (https://blast.ncbi.nlm.nih.gov/Blast.cgi), we found that *LcLTP* displayed a high homology with non-specific lipid-transfer protein 1 in *Medicago truncatula* (XP_003623613.3), soybean (XP_028213126.1), and *Cicer arietinum* (XP_004492552.1).

Plant *LTP*s are assumed to play important roles in membrane biogenesis, phospholipid transport, and biotic and abiotic stress response [27]. High *LTP* expression was associated with increased plant tolerance to cold, drought, and elevated hydrogen peroxide (H_2_O_2_) levels [28–30]. Peroxidases play prominent roles in antioxidant responses and stress tolerance in plants by catalysing H_2_O_2_ oxidoreduction, wherein they facilitate transfer of electrons from various donor molecules [16]. *PtrERF109* in *Poncirus trifoliate* was reported to contribute to cold tolerance by directly regulating the expression of *Prx1*, which is involved in antioxidative processes [31]. *LjGpxs1* and *LjGpxs3* perform major antioxidative functions in nodules, preventing lipid peroxidation and other oxidative processes at different subcellular sites of vascular and infected cells [32]. Collectively, *LTP* and *Prx* are considered to be important stress-responsive markers in scavenging ROS.

*RP* genes have been shown to be differentially regulated by environmental factors, both abiotic and biotic, which directly affect the plant growth and transcriptional regulation of *RP* genes and ultimately ribosome biogenesis. Transgenic rice overexpressing *RPL23A* conferred water usage and tolerance efficiency to drought and salt stresses [15]. Plant ribosomal proteins, RPL12 and RPL19, were reported to play a role in nonhost disease resistance against bacterial pathogens in *Nicotiana benthamiana* and *Arabidopsis* [33]. These reports indicated that *LcERF056* can enhance salt stress by regulating the *RP* gene.

**Fig. 7:**
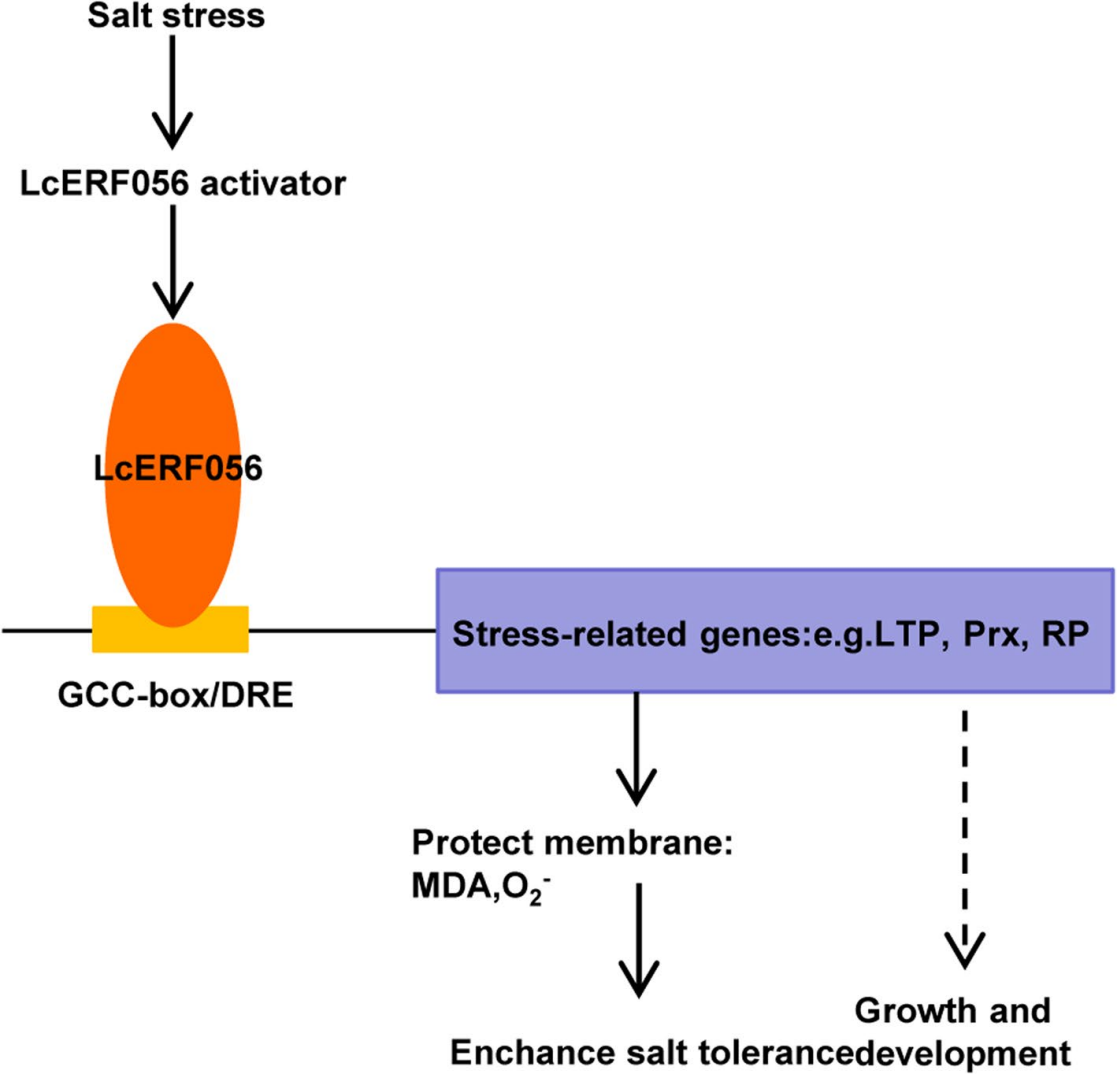
A proposed model describing the regulation mechanism of *LcERF056* in response to salt stress. LcERF056 activates the transcription of ROS-related genes such as *LcLTP* and *LcPrx* by directly binding to GCC-box or DRE element in the promoter and reducing reactive oxygen species, malondialdehyde (MDA), and relative electrolyte leakage. Therefore, it confers increased tolerance to salt stress in *L. corniculatus*

Based on the results, a functional model of *LcERF056* response to salt stress was proposed (Fig. 7). LcERF056 may have a unique value in its ability to confer salt tolerance to *L. corniculatus* and other plant species because of its considerable upregulation in response to salt. The key salt-responsive factor LcERF056 could directly upregulate ROS-related genes such as *LcLTP*, *LcPrx*, and *LcRP* by binding to *cis*-element GCC box or DRE element in the promoter and reducing ROS, MDA, and relative electrolyte leakage. In this way, it conferred increased tolerance to salt stress in *L. corniculatus*.

## Conclusions

In this study, the key salt-responsive *LcERF056* belonging to B3–1 (IX) subfamily was cloned and characterised, which was considerably upregulated by salt treatment and localised in nuclei as a transcriptional activator. Furthermore, *LcERF056*-OE transgenic *Arabidopsis* and *L. corniculatus* lines had significantly increased tolerance to salt treatment compared with WT or RNAi transgenic lines at the phenotypic and physiological levels. LcERF056 could bind to *cis*-element GCC box or DRE of ROS-related genes such as *LcLTP*, *LcPrx*, and *LcRP* as demonstrated through transcriptome analysis, ChIP-qPCR, and Y1H assay. These results suggested that the key regulator *LcERF056* plays important roles in salt tolerance by modulating ROS-related genes. Therefore, it may be a useful target for engineering salt-stress-tolerant *L. corniculatus* or other crops.

## Materials and methods

### Plant materials and growth conditions

The seeds of WT *L. corniculatus* L. cv Leo were purchased from Beijing Clover Seed & Turf Co. (Beijing, China), and *Arabidopsis thaliana* ecotype Columbia-0 were preserved in our laboratory. The plants were cultured at 25 ± 2 °C in a growth chamber with a photoperiod of 16 h/8 h (light intensity = 130–150 μE/m^2^/s and 50 μE/m^2^/s, respectively).

### Gene cloning and sequence analysis of *LcERF056*

The genomic and full cDNA sequences of *LcERF056* were amplified using the primers no. 1 (Table S1), and the PCR products were sequenced using ShengGong (Shanghai, China). A multiple alignment based on the amino acid sequences between LcERF056 and other ERF family members was performed using ClustalX2 [34]. Further, the phylogenetic tree was constructed using MEGA 5.0 [35]. The conserved domains and motifs were assessed using SMART [36].

### RNA extraction and expression pattern analysis

Total RNA was extracted from the seedings or different tissues of *L. corniculatus* by using an RNAprep Pure plant kit (Tiangen Biotech, Beijing, China). RT-PCR or qPCR was performed according to the procedure described in previous studies [11, 37]. The *LjUbi* gene (AW720576) was used as an internal standard. At least three biological replicates were used. All primer sequences are listed in Table S6.

### Subcellular localisation and transactivation assay of LcERF056

To construct the 35S::*LcERF056-GFP* expression plasmid, the CDS of the *LcERF056* was inserted into the binary vector p163-GFP. Both 35S::GFP and the recombinant vectors were transiently expressed in *Arabidopsis* protoplasts, as previously described [38]. Then the protoplasts were incubated in dark for at least 16 h. The GFP fluorescence was observed using a confocal laser scanning microscope (Zeiss LSM700).

*Saccharomyces cerevisiae* yeast strain AH109 and vector pDEST32 (Invitrogen, Carlsbad, CA) were used for studying the transactivation of *LcERF056*. The CDS of *LcERF056* was cloned into pDEST32 vector, and the constructed or empty plasmids were transformed into the yeast strain AH109 as per the manufacturer’s instructions (Clontech, Palo Alto, CA). The transformants were cultured on SD/−Leu and SD/−Leu/−His/−Ade + 3-Amino-1, 2, 4-triazole (3-AT) solid medium. β-Galactosidase filter-lifted test was measured according to the Yeast Protocols Handbook (Clontech).

### Plasmid construction and genetic transformation

The primers used in constructing vectors are listed in Supplemental Table S1. The full-length coding region of *LcERF056* was inserted into the binary vector pH7WG2D and pK7GW1WG2 to generate 35S:LcERF056 and RNAi-LcERF056 constructs, respectively. The obtained plasmid was then introduced into the *Agrobacterium tumefaciens* strain GV3101. *Agrobacterium* bearing the plasmids was transformed into *Arabidopsis* Col-0 plants by using the floral dip method [39]. The plasmids were introduced into *L. corniculatus* cotyledon by using *Agrobacterium*-mediated genetic transformation [40]. DNA or RNA was extracted from the transformed plants by using a DNA/RNA extraction kit (TIANGEN, Beijing, China), and PCR or qPCR was performed to identify the transgenic lines. Expression levels of *LcERF056* were calculated for different transgenic lines. Primer sequences used for PCR and qPCR are listed in Supplemental Table S2.

### Salt tolerance assays in *LcERF056* transgenic lines

Overall, 30-day-old soil-grown *LcERF056* transgenic *Arabidopsis* and the WT plants were irrigated with water containing 300 mM NaCl for 21 days and monitored during the next weeks. The experiment was repeated three times.

Stem cuttings of the transgenic *L. corniculatus* and WT were used for phenotypic, physiological, and biochemical analyses. When the adventitious roots were formed, the plants were transplanted to pots containing soil mixture (nutrient soil:vermiculite, 1:1, v/v) and grown at 22 °C with a photoperiod of 16 h/8 h (day/night) and 65% RH. Eight-week-old *LcERF056*-RNAi and *LcERF056*-OE transgenic lines were watered every 2 days with 250 mM NaCl for 25 days. As comparison, experiment using the WT as control plant was performed under similar conditions. All experiments were repeated three times.

### Measurements of the physiological and biochemical traits

The relative moisture content (%) was defined as (fresh weight − dry weight)/fresh weight × 100. Relative electrolyte leakage was calculated using the formula: (J2 − J1)/J2, where J1 is the conductivity of samples immersed in ddH_2_O for 2 h, and J2 is the conductivity of samples boiled for 10 min. The accumulation of O_2_^−^ was determined using nitro blue tetrazolium staining as per a method described previously [41]. Trypan blue staining [42] was used to visualise the degree of cell damage under salt stress. To assess the water status, the relative water content was determined following the method described by Liu et al. [43]. MDA content was determined according to the method described previously [44].

### RNA-Seq analysis

The penultimate three leaves were dissected from 8-week-old seedlings from WT, RNAi, and OE lines. Samples from five siblings were pooled. Three biological repeats of RNAi lines (RNAi-4, RNAi-8, and RNAi-11) and OE lines (OE-9, OE-12, OE-16) were performed per genotype. Total RNA was isolated from each sample, and RNA-Seq was performed at The Beijing Genomics Institute (BGI) (Beijing, China). The differentially expressed genes were from different groups. WT-VS-OE and WT-VS-RNAi indicated the plants with *LcERF056* overexpression and RNAi *LcERF056* compared with WT plants, respectively.

### Y1H assay

Y1H assays were performed using the Matchmaker Gold Yeast One-Hybrid System (Clontech). Three tandem copies of the fragment containing GCC box of the four detection promoters were used as the bait in the Y1H screens (Table S7). We generated the construct of bait with GCC box of the detection promoter in front of the reporter gene AUR1-C and construct prey of *LcERF056* with antibiotic resistance gene that conferred AbA (400 ng/mL) resistance to yeast.

### ChIP-qPCR

ChIP experiments were performed as previously described, with minor modifications (Kaufmann and Al. 2010). Specific antibodies targeting LcERF056 were synthesised (GenScript, Shanghai, China). In brief, 8-week-old seedlings (WT, RNAi, OE) were cross-linked with 1% formaldehyde. WT plants were used as control. The chromatin DNA fragments were isolated from their nuclei to retrieve binding DNA fragments by using rabbit polyclonal antibodies with the Magna ChIPTM A kit (Millipore). The bound DNA fragments were extracted using the Gel/PCR DNA fragments extraction kit (Millipore). The levels of the bound DNAs were measured through qPCR. Primer sequences are given in Supplemental Table S7.

### Statistical analysis

The data were analysed using SPSS version 17.0. Means and standard errors were calculated to compare variables. The least significant difference test at *P* < 0.05 or < 0.01 was performed to consider statistical significance.

## Supplementary Information

**Additional file 1 **Fig. S1 Sequence analysis of *LcERF056* from *L. corniculatus*. **(A)** Nucleotide and deduced amino acid sequences of the LcERF056 protein. The conserved AP2 domain was underlined. **(B)** The Neighbour-Joining phylogenetic tree of LcERF056 and other ERF members in plants. The complete amino acid sequences were aligned with ClustalX2, and the phylogenetic tree was constructed using the MEGA 5.0 software with 1000 bootstrap replicates. The accession number of each appended protein is as follows (in parentheses): OsERF001 (Os06g40150.1), AtERF004 (AT5G11190), GmERF083 (TC431280), OsERF098 (Os02g34260.1), LcERF056 (KC777345), AtRAP2.6 (AT1G43160), PtERF (eugene3.00031319), VvERF (GSVIVP00006201001), AtERF013 (AT2G44840), AtERF073 (AT1G72360), OsERF059 (Os10g25170.1), OsERF074 (Os05g41780.1), AtERF078 (AT3G15210), OsERF083 (Os03g64260.1), AtERF101 (AT5G47220), OsERF053 (Os01g12440.1), AtERF064 (AT4G23750). **(c)** Comparison of the deduced LcERF056 and AtERF13 proteins. **Fig. S2** Genomic PCR and semi quantitative RT-PCR in transgenic *L. corniculatus*. **(A)** *LcERF056*-OE vector and *LcERF056*-RNAi vector. **(B)** Transgenic *LcERF056*-OE lines were confirmed by checking genes *LcERF056* and hygromycin using genomic PCR. The forward primer was from 35S sequence of vector, and reverse primer was from *LcERF056* sequence; **(C)** Transgenic *LcERF056*-RNAi lines were confirmed by checking genomic PCR. The forward primer was from intron sequence of vector, and the reverse primer was from *LcERF056* sequence; **(D)** Semi quantitative RT-PCR of *LcERF056* in OE and RNAi transgenic lines. **Fig. S3** Morphological comparison of *L. corniculatus* wild-type (WT) vs. *LcERF056*-OE and *LcERF056*-RNAi transgenic plants. **(A)** qPCR analysis of *LcERF056* expression in the WT and transgenic lines. **(B)** Phenotype analysis of height and branches of WT, *LcERF056*-OE, and *LcERF056*-RNAi lines. Bars = 5 cm. **(C)** Plant height. **(D)** Branch number. *P* < 0.05 indicated significant difference between WT and transgenic lines. **Fig. S4** The GO bar charts of DEGs in RNA-Seq data. **(A)** The numbers of DEGs in *LcERF056*-OE VS WT, and *LcERF056*-RNAi VS WT, and *LcERF056*-OE VS *LcERF056*-RNAi. **(B)** The GO terms of DEGs between transgenic and WT. **Fig. S5** LcERF056-antibody in *LcERF056*-RNAi, WT and *LcERF056*-OE. **Table S1.** Primers used for constructing vector. **Table S2.** Primers used for detecting transgenic *L. corniculatus.* **Table S3.** List of the 18 overlapped genes. **Table S4.** The GO terms of 18 overlapped genes. **Table S5.** Information on the primers of genes used in qPCR reactions. **Table S6.** GCC-box in the promoters. **Table S7.** 4 × GCC or 4 × DRE in the Y1H **Table S8.** Primer sequences used in ChIP-qPCR

## Data Availability

All LcERFs and targets sequence information is available in the *Lotus japonicus* Genomics Database (http://www.kazusa.or.jp/lotus/). The data generated or analyzed during current research are included in this published article and its supplemental data files and available from the corresponding author on reasonable request.

## Abbreviations

ROS: Reactive oxygen species
AP2/ERF: APETALA2/ethylene response factor
TF: Transcription factor
OE: Overexpression
RNAi: RNA interference
qPCR: Quantitative real-time Polymerase Chain Reaction
*LTP*: Lipid-transfer protein.
WT: Wild-type
*Prx*: Peroxidase
*RP*: Ribosomal protein
GFP: Green fluorescent protein
Y1H: Yeast one-hybrid
MDA: Malondialdehyde
GO: Gene ontology
AbA: Aureobasidin A
ChIP: Chromatin immunoprecipitation
NBT: Nitro blue tetrazolium
RWC: Relative water content
