# Supplementary material for: The key regulator LcERF056 enhances salt tolerance by modulating reactive oxygen species-related genes in Lotus corniculatus
Source: BMC Plant Biol. 2021 Dec 29;21:605. doi: 10.1186/s12870-021-03336-4 (PMC8715585; doi:10.1186/s12870-021-03336-4)
Supplement: Supplementary file 1 — Additional file 1 Fig. S1 Sequence analysis of LcERF056 from L. corniculatus. (A) Nucleotide and deduced amino acid sequences of the LcERF056 protein. The conserved AP2 domain was underlined. (B) The Neighbour-Joining phylogenetic tree of LcERF056 and other ERF members in plants. The complete amino acid sequences were aligned with ClustalX2, and the phylogenetic tree was constructed using the MEGA 5.0 software with 1000 bootstrap replicates. The accession number of each appended protein is as follows (in parentheses): OsERF001 (Os06g40150.1), AtERF004 (AT5G11190), GmERF083 (TC431280), OsERF098 (Os02g34260.1), LcERF056 (KC777345), AtRAP2.6 (AT1G43160), PtERF (eugene3.00031319), VvERF (GSVIVP00006201001), AtERF013 (AT2G44840), AtERF073 (AT1G72360), OsERF059 (Os10g25170.1), OsERF074 (Os05g41780.1), AtERF078 (AT3G15210), OsERF083 (Os03g64260.1), AtERF101 (AT5G47220), OsERF053 (Os01g12440.1), AtERF064 (AT4G23750). (c) Comparison of the deduced LcERF056 and AtERF13 proteins. Fig. S2 Genomic PCR and semi quantitative RT-PCR in transgenic L. corniculatus. (A) LcERF056-OE vector and LcERF056-RNAi vector. (B) Transgenic LcERF056-OE lines were confirmed by checking genes LcERF056 and hygromycin using genomic PCR. The forward primer was from 35S sequence of vector, and reverse primer was from LcERF056 sequence; (C) Transgenic LcERF056-RNAi lines were confirmed by checking genomic PCR. The forward primer was from intron sequence of vector, and the reverse primer was from LcERF056 sequence; (D) Semi quantitative RT-PCR of LcERF056 in OE and RNAi transgenic lines. Fig. S3 Morphological comparison of L. corniculatus wild-type (WT) vs. LcERF056-OE and LcERF056-RNAi transgenic plants. (A) qPCR analysis of LcERF056 expression in the WT and transgenic lines. (B) Phenotype analysis of height and branches of WT, LcERF056-OE, and LcERF056-RNAi lines. Bars = 5 cm. (C) Plant height. (D) Branch number. P < 0.05 indicated significant difference between WT and transgenic lines. Fig. S4 The GO [file 12870_2021_3336_MOESM1_ESM.docx]

Supplemental file (figures and tables)

**
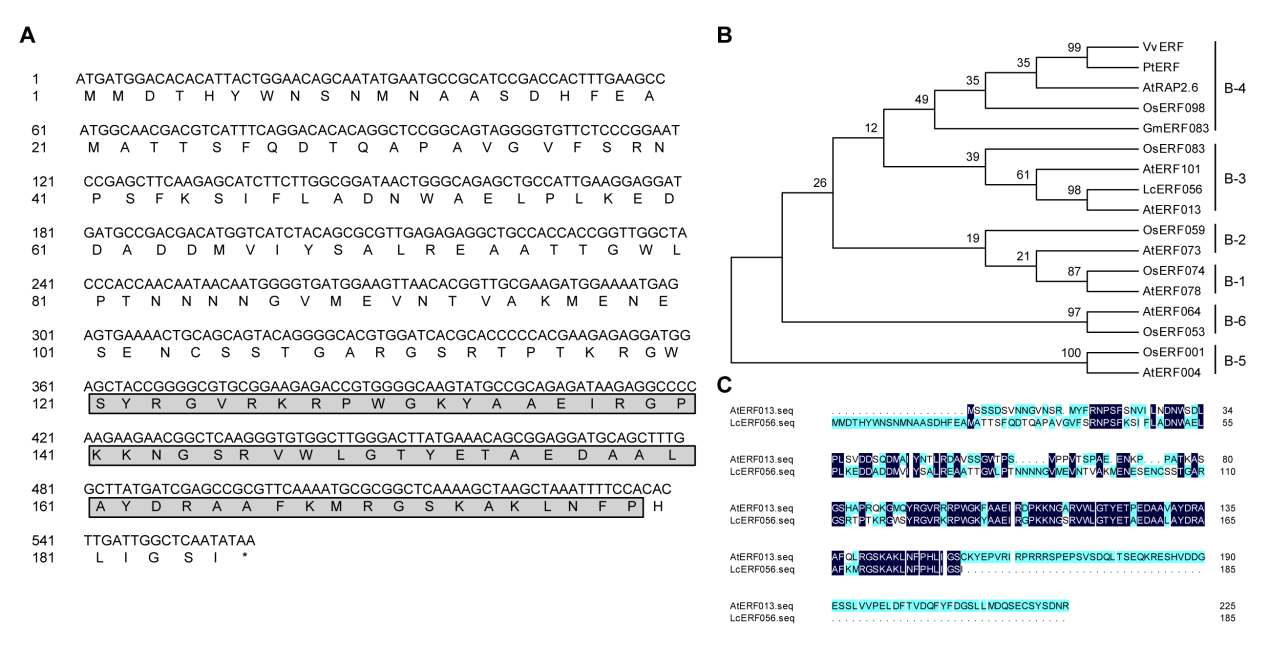
**

**Fig. S1** **a,** Nucleotide and deduced amino acid sequences of the LcERF056 protein. The conserved AP2 domain is underlined. **b,** The phylogenetic tree of LcERF056 and other ERF proteins in plants. The complete amino acid sequences of LcERF056 and other ERF proteins were aligned by ClustalX2, and the phylogenetic tree was constructed by neighbor-joining algorithms of the MEGA 5.0 software. The accession number of each appended protein is as follows (in parentheses):OsERF001 (Os06g40150.1), AtERF004 (AT5G11190), GmERF083 (TC431280), OsERF098 (Os02g34260.1), LcERF056 (KC777345), AtRAP2.6 (AT1G43160), PtERF (eugene3.00031319), VvERF (GSVIVP00006201001), AtERF013 (AT2G44840), AtERF073 (AT1G72360), OsERF059 (Os10g25170.1), OsERF074 (Os05g41780.1), AtERF078 (AT3G15210), OsERF083 (Os03g64260.1), AtERF101 (AT5G47220), OsERF053 (Os01g12440.1), AtERF064 (AT4G23750). **(C)** Comparison of the deduced LcERF056 and AtERF13 proteins.


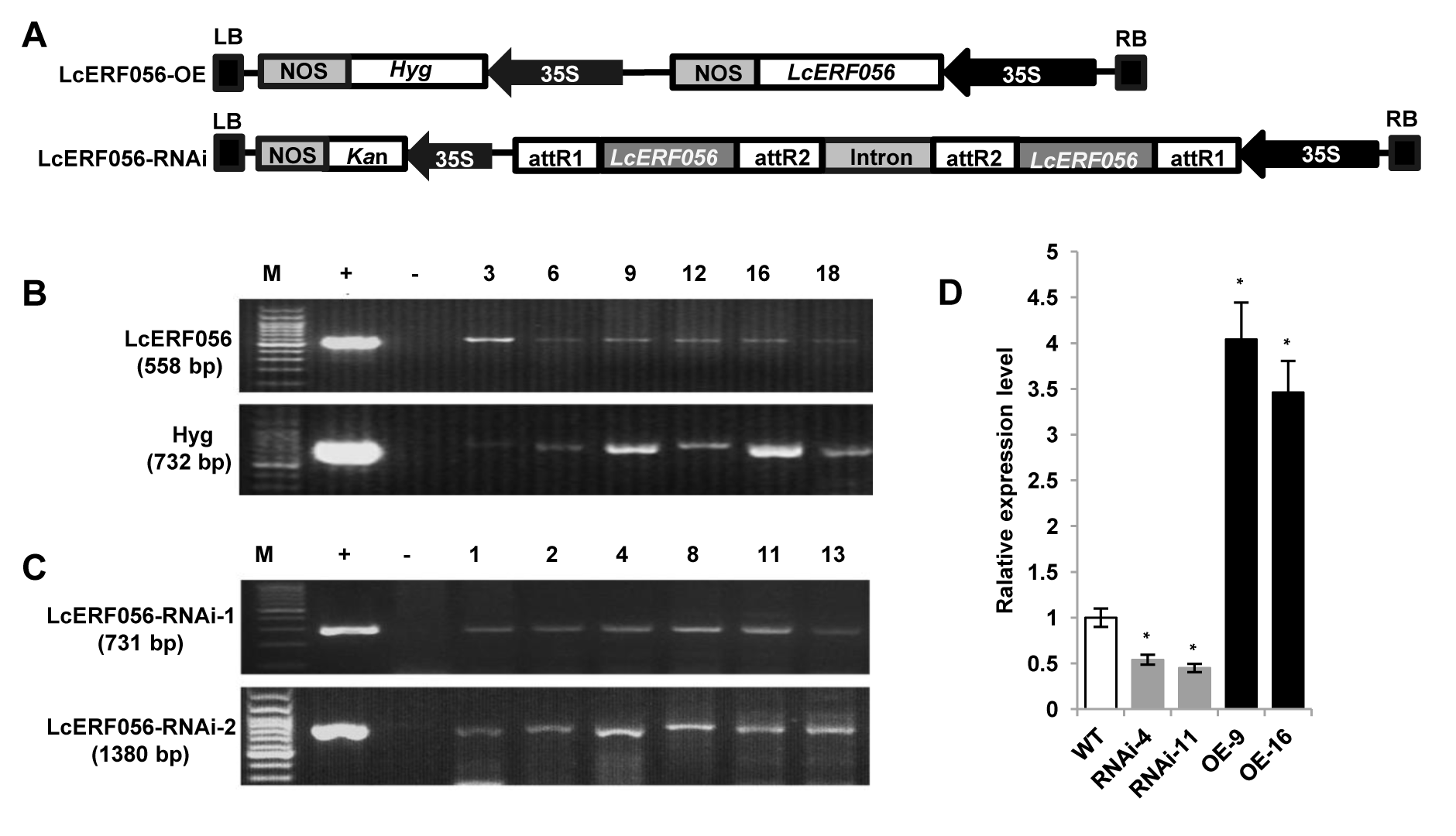


**Fig. S2** **Identiﬁcation and validation of *LcERF056* transgenic *lotus corniculatus*. a,** Constructs used to overexpress and RNA interference expression *LcERF056*. **b,** Identification of *LcERF056*-OE transgenic plantlets by checking genes *LcERF056* and *hygromycin* (*Hyg*) using genomic PCR with specific primers for the vector (Table S2). M: DNA marker; +: positive vector; -: wild type; **c,** Identification of *LcERF056*-RNAi transgenic plantlets by genomic PCR with specific primers for the vector (Table S2). M: DNA marker; +: positive vector; -: wild type; **d,** qPCR-determined expression levels of two representative *LcERF056*-OE and *LcERF056*-RNAi transgenic lines. Error bars indicate SD values from three independent biological replicates.

**
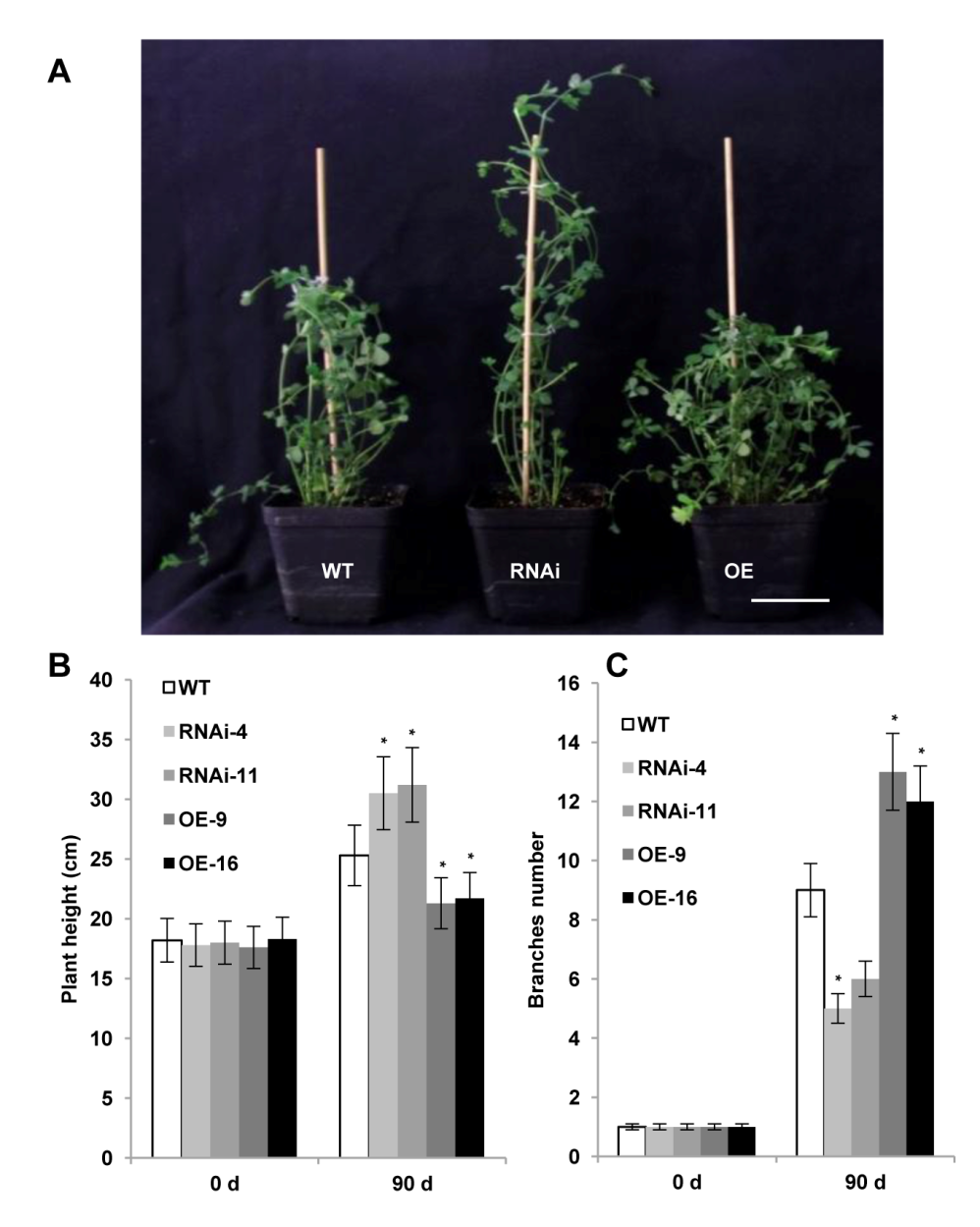
**

**Fig. S3** Morphological comparison of *L. corniculatus* wild-type vs. *LcERF056*-OE and *LcERF056* -RNAi transgenic plants. **a,** Phenotype analysis of height and branches of WT, *LcERF056*-OE and *LcERF056*-RNAi lines. Bars=5 cm. **b,** Plant height. **c,** Branches number. Significant difference between WT and transgenic Lines (P <0.05).

**
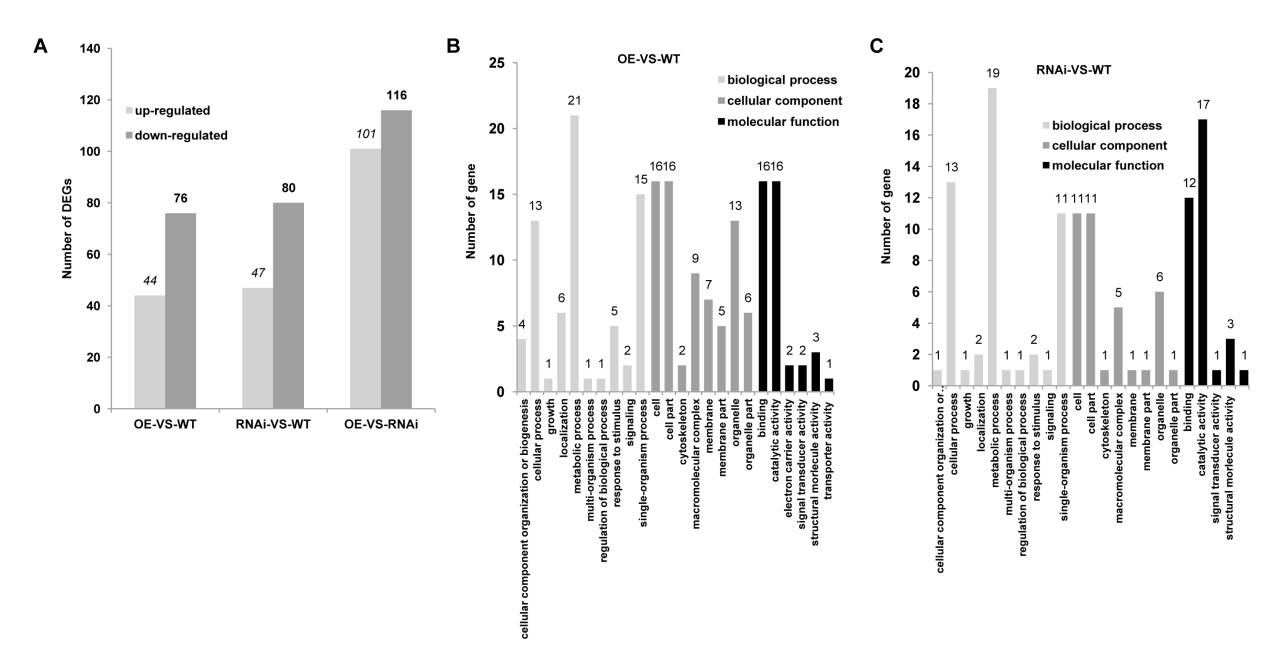
**

**Fig. S4 a,** The GO bar charts of DGEs in RNA-Seq data. **b,** The numbers of DEGs in *LcERF056*-OE VS WT, and *LcERF056*-RNAi VS WT, and *LcERF056*-OE VS *LcERF056*-RNAi. **c,** The GO terms of DEGs between transgenic and WT.


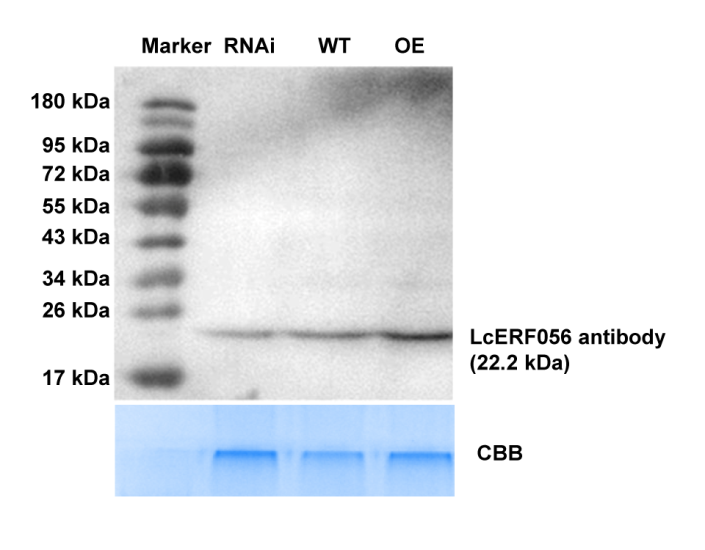


**Fig. S5** LcERF056-antibody in *LcERF056*-RNAi, WT and *LcERF056*-OE.

**Table S1.** Primers of constructing vector

| Serial | Plasmid | Primer name | Sequence (5' to 3') |
| --- | --- | --- | --- |
| 1 | PEASY-T | LcERF056-F | ATGATGGACACACATTACTGGAAC; |
|  |  | LcERF056-R | TTATATTGAGCCAATCAAGTGTGG; |
| 2 | p163-GFP | SalI, BamHI | CGCGTCGACATGATGGACACACATTAC; |
|  |  |  | TATGGATCCCACTTGATTGGCTCAATA; |
| 3 | pET32a | *Bam*HI, *Sal*I | GGGGATCCATGATGGACACACATTAC; |
| 4 | pAD gal4-2.1 |  | TATGTCGACTTATATTGAGCCAATCAA; |
| 5 | pBridge | *EcoRI, BamHI* | GAATTCATGATGGACACACATTACTG; |
|  |  |  | GGATCCTTATATTGAGCCAATCAAGTG; |
| 6 | pDONR201 | *attB1, attB2* | GGGGACAAGTTTGTACAAAAAAGCAGGCTGC +LcERF056-F; |
|  |  |  | GGGGACCACTTTGTACAAGAAAGCTGGGT +LcER056-R; |
| 7 | pDEST32 | *attR1, attR2* | LR reaction; |
| 8 | pH7WG2D | *attR1, attR2* | LR reaction; |
| 9 | pK7GW1WG2 | Intron-F | CCTTACCTCATCATTTCCATG; |
|  |  | Intron-R | CATGGAAATGATGAGGTAAGG; |

**Table S2.** Primers of detecting transgenic *Lotus corniculatus*

| Serial | Transgenic line | Primer name | Sequence (5' to 3') |
| --- | --- | --- | --- |
| 1 | *LcERF056*-OE | 35S-F | GACGCACAATCCCACTATCC; |
|  |  | LcERF056-R | TTATATTGAGCCAATCAAGTGTGG; |
| 2 | Hgy | Hgy-F | CTGCTCCATACAAGCCA; |
|  |  | Hgy-R | ATGAAAAAGCCTGAACTC; |
| 3 | *LcERF056*-RNAi-1 | LcERF056-F | ATGATGGACACACATTACTGGAAC; |
| 4 |  | Intron-R | CATGGAAATGATGAGGTAAGG; |
| 5 | *LcERF056*-RNAi-2 | Intron-F | CCTTACCTCATCATTTCCATG; |
|  |  | LcERF056-R | TTATATTGAGCCAATCAAGTGTGG; |

**Table S3.** List of the 18 overlapped genes**.**

| Serial | GeneID | Gene name | OE | Ratio(Log2) | P_value | RNAi | Ratio(Log2) | P_value |
| --- | --- | --- | --- | --- | --- | --- | --- | --- |
| 1 | Lj0g3v0296809 | lipid transfer protein (LTP2) | Up | 4.99 | 0.76 | Down | -4.43 | 0.32 |
| 2 | Lj0g3v0342769 | Lipoxygenase (LOX) | Up | 4.44 | 0.82 | Down | -0.56 | 0.27 |
| 3 | Lj0g3v0342749 | Lipoxygenase (LOX2) | Up | 4.13 | 0.72 | Down | -1.20 | 0.25 |
| 4 | Lj0g3v0053229 | Glucose-6-phosphate isomerase (GPI) | Up | 4.063 | 0.77 | Down | -0.84 | 0.27 |
| 5 | Ljchlorog3v0015670 | Glucose-6-phosphate isomerase (GPI) | Up | 4.063 | 0.77 | Down | -0.84 | 0.27 |
| 6 | Lj1g3v4955450 | Calmodulin (CaM) | Up | 3.87 | 0.74 | Down | -2.65 | 0.39 |
| 7 | Lj4g3v2678550 | RRP12-like protein (RRP12-L) | Up | 3.24 | 0.7554 | Down | -1.14 | 0.42 |
| 8 | Lj6g3v1933980 | Ribosomal protein S14 e(RpS) | Up | 3.0076 | 0.7299 | Down | -8.336 | 0.77 |
| 9 | Lj6g3v1953890 | Glutamine synthetase (GS) | Up | 2.123 | 0.703 | Down | -11.481 | 0.955 |
| 10 | Lj0g3v0325029 | Polymerase II subunit RPB1 (RPB1) | Up | 2.0149 | 0.669 | Down | -9.674 | 0.8799 |
| 11 | Lj0g3v0128239 | Lipid-transfer protein (LTP) | Up | 2.97 | 0.62 | Down | -10.33 | 0.91 |
| 12 | Lj0g3v0191189 | Asparagine synthetase (ASNS) | Up | 1.51 | 0.39 | Down | 0.005 | 0.89 |
| 13 | Lj0g3v0242029 | Ribosomal protein (RP) | Up | 1.55 | 0.38 | Down | 0.001 | 0.88 |
| 14 | Lj4g3v0768720.1 | Peroxidase (Prx) | Up | 1.16 | 0.26 | Down | 0.0003 | 0.97 |
| 15 | Lj3g3v3364600 | Transmembrane protein | Up | 0.098 | 0.202 | Down | -9.73 | 0.88 |
| 16 | Lj1g3v0112720 | Dioxygenase | Down | -0.027 | 0.163 | Up | 7.051 | 0.92 |
| 17 | Lj0g3v0145139 | Lectin protein | Down | -0.95 | 0.22 | Up | 6.16 | 0.89 |
| 18 | Lj1g3v4515810 | Peptide transporter | Down | -0.93 | 0.28 | Up | 4.76 | 0.82 |

**Table S4.** The GO terms of 18 overlapped genes**.**

| Serial | GeneID | GO Component | GO Function | GO Process |
| --- | --- | --- | --- | --- |
| 1 | Lj0g3v0296809 | — | — | — |
| 2 | Lj0g3v0342769 | — | — | metabolic process |
| 3 | Lj0g3v0342749 | GO:0044424 | oxidoreductase activity | fatty acid biosynthetic process |
| 4 | Lj0g3v0053229 | — | — | — |
| 5 | Ljchlorog3v0015670 | — | — | — |
| 6 | Lj1g3v4955450 | — | — | — |
| 7 | Lj4g3v2678550 | — | — | — |
| 8 | Lj6g3v1933980 | intracellular ribonucleoprotein complex | structural molecule activity | gene expression |
| 9 | Lj6g3v1953890 | plastid | adenyl ribonucleotide binding | glutamine metabolic process |
| 10 | Lj0g3v0325029 | — | — | — |
| 11 | Lj0g3v0128239 | — | — | establishment of localization |
| 12 | Lj0g3v0191189 | — | carbon-nitrogen ligase activity | glutamine family amino acid metabolic process |
| 13 | Lj0g3v0242029 | intracellular ribonucleoprotein complex | structural molecule activity | gene expression |
| 14 | Lj4g3v0768720.1 | — | — | — |
| 15 | Lj3g3v3364600 | — | — | — |
| 16 | Lj1g3v0112720 |  | oxidoreductase activity | GO:0043102 |
| 17 | Lj0g3v0145139 | — | — | — |
| 18 | Lj1g3v4515810 | GO:0044464 | — | peptide transport |

**Table S5.** Information on the primers of genes used in qPCR reactions

| Gene name | Gene ID | Primer name | Sequence (5' to 3') |
| --- | --- | --- | --- |
| LcERF056 | KC777345 | LcERF056-F | TGCCGCATCCGACCACTT |
|  |  | LcERF056-R | TTCCATCTTCGCAACCGT |
| Polyubiquitin | AW720576 | LcUbi-F | CAAGGAAGGTATCCCACCG |
|  |  | LcUbi-R | TTAGAATCCACCACGAAGACG |
| Lipid transfer protein (LTP2) | Lj0g3v0296809 | LcLTP2-F | TGGCTTGCTTCAATCTTT |
|  |  | LcLTP2-R | GCACTACTTCCCTTGTCACTT |
| Lipoxygenase(LOX) | Lj0g3v0342769 | LcLOX-F | GACCCCCATTTTCCACCTC |
|  |  | LcLOX-R | TATTGTGCTTGTGTGATCCCC |
| Lipoxygenase(LOX2) | Lj0g3v0342749 | LcLOX2-F | TAGATCACCACGACACGA |
|  |  | LcLOX2-R | AAGCCTTTGCCAGTAGCC |
| polymerase II subunit (RPB1) | Lj0g3v0325029.1 | LcRPB1-F | ATACAAATACCCTTCACC |
|  |  | LcRPB1-R | TATGGTTTCTTTGCTGGA |
| Calmodulin (CaM) | Lj1g3v4955450.1 | LcCaM-F | TGTGCTCCTCCTGTTTCC |
|  |  | LcCaM-R | TCTGTTGCGATCCCTGTC |
| Asparagine synthetase (ASN) | Lj0g3v0191189 | LcASN-F | GATTGTGAACATTTTGAGGTC |
|  |  | LcASN-R | AGTAGCGAGAAGTGATGGAAG |
| Ribosomal RNA processing (RRP12-like) | Lj4g3v2678560 | LcRRP12L-F | CACCTGCTCTACCATCAACC |
|  |  | LcRRP12L-R | AACAGCCACCATCATCTTTT |
| Lipid-transfer protein(LTP) | Lj0g3v0128239 | LcLTP-F | AACGGAGCTTAACAGAAGGG |
|  |  | LcLTP-R | TCAGAAATGAGCGATCGGGG |
| Ribosomal protein(RP) | Lj0g3v0242029 | LcRP-F | GGGTTTGTGAGGTAGGAGA |
|  |  | LcRP-R | CTCATTAGGGACAAGCCAGA |
| Peroxidase(Prx) | Lj4g3v0768720 | LcPrx-F | TTAGAAATTGGGAACTTGTG |
|  |  | LcPrx-R | TGGCTCTGAAAGGGACAT |

**Table S6.** GCC-box in the promoters

| Gene | Gene ID | Cis-element | Location in the promoter | consensus |
| --- | --- | --- | --- | --- |
| LcLTP (Lipid-transfer protein) | Lj0g3v0128239 | DRE/CRT-element | -524~-520 | CCGAC |
| LcRP(Ribosomal protein) | Lj0g3v0242029.1 | GCC-box | -162~-156 | GCCGCC |
| LcPrx (Peroxidase) | Lj4g3v0768720.1 | GCC-box | -598~-593 | GGCGGC |

**Table S7.** 4×GCC or 4×DRE in the Y1H

| Gene | Gene ID |  |
| --- | --- | --- |
| LcLTP (Lipid-transfer protein) | Lj0g3v0128239 | LcLTP4×DRE-F：  AGCTTGGGGCCCCGACCGCTGGGGCCCCGACCGCTGGGGCCCCGACCGCTGGGGCCCCGACCGCTC；  LcLTP4×DRE -R：  TCGAGAGCGGTCGGGGCCCCAGCGGTCGGGGCCCCAGCGGTCGGGGCCCCAGCGGTCGGGGCCCCA;  LcLTP4×mDRE-F：  AGCTTGGGGCCTTTTCCGCTGGGGCCTTTTCCGCTGGGGCCTTTTCCGCTGGGGCCTTTTCCGCTC；  LcLTP4×mDRE-R：  TCGAGAGCGGAAAAGGCCCCAGCGGAAAAGGCCCCAGCGGAAAAGGCCCCAGCGGAAAAGGCCCCA; |
| LcRP(Ribosomal protein) | Lj0g3v0242029.1 | LcRP4×GCC-F:  AGCTTTAAGGTGCCGCCAAGTAAGGTGCCGCCAAGTAAGGTGCCGCCAAGTAAGGTGCCGCCAAGC;  LcRP4×GCC-R:  TCGAGCTTGGCGGCACCTTACTTGGCGGCACCTTACTTGGCGGCACCTTACTTGGCGGCACCTTAA;  LcRP4×mGCC-F:  AGCTTTAAGGTTCCTCCAAGTAAGGTTCCTCCAAGTAAGGTTCCTCCAAGTAAGGTGCCGCCAAGC;  LcRP4×mGCC-R:  TCGAGCTTGGAGGAACCTTACTTGGAGGAACCTTACTTGGAGGAACCTTACTTGGAGGAACCTTAA; |
| LcPrx (Peroxidase) | Lj4g3v0768720.1 | LcPrx4×GCC-F:  AGCTTGACAAAGGCGGCTATGACAAAGGCGGCTATGACAAAGGCGGCTATGACAAAGGCGGCTATC;  LcPrx4×GCC-R:  TCGAGATAGCCGCCTTTGTCATAGCCGCCTTTGTCATAGCCGCCTTTGTCATAGCCGCCTTTGTCA;  LcPrx4×mGCC-F:  AGCTTGACAAAGGAGGATATGACAAAGGAGGATATGACAAAGGAGGATATGACAAAGGAGGATATC;  LcPrx4×mGCC-R:  TCGAGATATCCTCCTTTGTCATATCCTCCTTTGTCATATCCTCCTTTGTCATATCCTCCTTTGTCA; |

**Table S8.** Primer sequences used in ChIP-qPCR

| Gene | Gene ID | Primmer name | Sequence (5' to 3') |
| --- | --- | --- | --- |
| LcLTP (Lipid-transfer protein) | Lj0g3v0128239 | LcLTP-pro-F | AACGGAGCTTAACAGAAGGG |
|  |  | LcLTP-pro-R | TCAGAAATGAGCGATCGGGG |
| LcRP(Ribosomal protein) | Lj0g3v0242029.1 | LcRP-pro-F | GGGTTTGTGAGGTAGGAGA |
|  |  | LcRP-pro-R | CTCATTAGGGACAAGCCAGA |
| LcPrx (Peroxidase) | Lj4g3v0768720.1 | LcPrx-pro-F | TTAGAAATTGGGAACTTGTG |
|  |  | LcPrx-pro-R | TGGCTCTGAAAGGGACAT |
